# Household Wealth and Individuals’ Mental Health: Evidence from the 2012–2018 China Family Panel Survey

**DOI:** 10.3390/ijerph191811569

**Published:** 2022-09-14

**Authors:** Rui Zhang, Chenglei Zhang, Jiahui Xia, Dawei Feng, Shaoyong Wu

**Affiliations:** 1 Department of Economics, Jinan University, Guangzhou 510632, China; 2 School of Economics & Trade, Guangdong University of Finance, Guangzhou 510521, China; 3 School of Management, Jinan University, Guangzhou 510632, China; 4 Institute of Industrial Economics, Jiangxi University of Finance and Economics, Nanchang 330013, China

**Keywords:** household wealth, mental health, insurance investment, labor supply, medium- and long-term impact

## Abstract

Based on the data from the 2012–2018 China Family Panel Survey, this study examines the impact of household wealth on individuals’ mental health using a two-way fixed effects model. The findings indicate that household wealth exerts a significant positive effect on individuals’ mental health. Furthermore, this study shows that the impact of household wealth on individuals’ mental health is nonlinear but inverted U-shaped. Considering the possible endogeneity problem, this study further examines the effect of household wealth on residents’ mental health using two-stage least squares, and the conclusions remain robust. The results of the heterogeneity analysis indicate that household wealth has a greater impact on the mental health of residents in the low-education group and western region. Furthermore, the results of the mechanisms reveal that household wealth affects mental health by influencing insurance investment and individuals’ labor supply. Moreover, this study finds that household wealth affects individuals’ mental health not only in the short term but also in the medium and long terms. This study provides policy implications for the government toward improving individuals’ mental health.

## 1. Introduction

Traditionally, mental health has been an important issue in both developing and developed countries. From a macroscopic perspective, mental health, as an extremely important human capital, is of great importance to the economic development of a country. Meanwhile, the improvement of health status is crucial to the progress of human civilization and sustainable socioeconomic development. From a microscopic perspective, the mental health status of a population affects labor supply and individuals’ quality of life [1,2]. Additionally, severe psychological problems may lead to suicide. Data from the World Health Organization suggest that approximately 703,000 people worldwide die by suicide annually [3]. In summary, improving the populations’ mental health is important for promoting individuals’ welfare as well as facilitating stable socioeconomic development.

Given that health might affect the well-being of a population, numerous studies have examined the determinants of health from different perspectives. Previous studies have found that the natural environment [4,5,6,7], macroeconomic fluctuations [8,9], and level of corruption in a country [10] can affect the mental health of a population. Although many factors at the macro level can affect individuals’ health, some factors at the household and individual levels that also affect health cannot be ignored. Studies at the household and individual levels have found that personal religious beliefs [11], social capital [12], and unemployment [13] affect residents’ health. While income is an important guarantee for individuals’ clothing, food, housing, and transportation, which are necessary for most people to survive, its impact on health has been a common focus of economists and sociologists [14,15,16,17]. Most studies support the conclusion that individual income positively affects the individuals’ mental health.

Wealth, compared to income, represents a person’s lifetime reserve of financial resources, and it has a crucial impact on individuals. It has been shown that wealth is more stable than income [18,19], and it can buffer the effects of loss of income or temporary low income, as well as function as a better measure of the economic status of individuals at different stages of their lives. Thus, wealth is a more accurate measure of an individual’s actual economic level [20,21]. In addition, relative to other factors that measure an individual’s socioeconomic status, wealth serves as a better means. Moreover, high-wealth groups are more likely to have access to political power, social prestige, socioeconomic status, education, and employment opportunities [21,22,23]. In summary, the impact of wealth on health may be more profound than that of income. However, current research has mainly focused on the income perspective, and only a few studies have examined the impact of household wealth on the mental health of Chinese residents. In view of the richness of previous studies on the effects of income on residents’ health and considering that the effects of wealth on health may differ from those of income, this study examines the effects of household wealth on Chinese residents’ mental health.

The main contributions of this study are as follows: first, most previous studies focus on the short-term effects of household wealth on individuals’ mental health, but this study examines not only the short-term effects but also the medium- and long-term effects. Additionally, different from previous studies, this study not only examines the impact of accumulated wealth on individuals’ mental health, but also investigates the effects of changes in household wealth on individuals’ health. Second, this study examines the heterogeneity of the effects of household wealth on the mental health of individuals with different levels of education and occupying diverse regions, which facilitates the understanding of the heterogeneous effects of wealth on mental health. Third, this study clarifies the mechanism of the effect of household wealth on individuals’ mental health, which provides policy implications for governments to improve individuals’ mental health.

## 2. Theoretical Analysis and Hypothesis

Household wealth can influence individuals’ mental health in different ways. Household wealth can provide individuals with basic needs such as food, basic medical care, and housing. In terms of living environment, it varies significantly among individuals with different levels of wealth. Individuals who have accumulated more wealth have better living environments, more convenient transportation facilities, as well as improved medical services, convenience stores, and other basic living resources. Individuals who lack accumulated wealth often have poor living environments, and the level of healthcare, street security, or other infrastructure is relatively low. This may reduce their life satisfaction and happiness, and thus affect their mental health. Additionally, household wealth can improve individuals’ ability to withstand negative shocks. Inevitably, negative shocks occur in life (e.g., unemployment, natural disasters, economic crises, and the coronavirus disease 2019 (COVID-19) pandemic). Therefore, individuals who lack wealth accumulation attributes may face greater financial stress, which may lead to negative emotions, cardiovascular and immune system threats, and further negative effects on the health of the population [24,25]. Meanwhile, many studies have found that individuals in the lower percentile of wealth levels may suffer from stigmatization and discrimination, which subsequently affect their mental health [18,22,26,27]. In summary, this study proposes the following hypothesis:

**Hypothesis** **1.** *Household wealth will significantly increase individual mental health.*

Household wealth may increase an individuals’ investment in health insurance. The group at the top of the wealth chain has more concentrated resources and fortunes and is more likely to have access to various health insurance schemes and services. For those affluent groups, an increase in the price of health services, such as health insurance, can lead to a lack of material resources to maintain health, which is detrimental to accessibility to health insurance products and health services [28,29]. Meanwhile, the wealthy have higher social capital and can swiftly obtain more relevant information about health investments. Information-related advantages and social capital facilitate the purchase of health insurance by the rich. The poor often have difficulty or are burdened by high costs vis à vis obtaining basic health insurance due to information asymmetry. In conclusion, household wealth may dramatically increase the likelihood of an individual or household purchasing health insurance.

Health insurance may have a positive impact on an individual’s mental health, and it is an important component of healthcare investment. The direct function of health insurance is to guarantee the financial accessibility of healthcare utilization in the case of illness, which can largely relieve the financial pressure of patients to seek medical treatment and increase the use of health services. Therefore, purchasing health insurance may have a positive impact on the health of a population. Previous studies have also validated this view [30,31]. Thus, we formulate the following hypothesis:

**Hypothesis** **2.** *Household wealth can improve individuals’ mental health by increasing their investment in health insurance.*

Household wealth can have a significant negative effect on individuals’ labor supply. Based on social comparison theory, people possess social attributes, and individuals make social comparisons spontaneously, consciously, or unconsciously. Different families have varying levels of wealth, and individuals inevitably compare their household wealth with the accumulation of wealth of their surrounding groups. The results of the comparison inevitably affect an individuals’ self-perception and evaluation. If a family’s wealth is much smaller than that of the surrounding group, the individual feels a sense of relative deprivation of wealth. From a social psychology perspective, an increased sense of deprivation may cause individuals to experience negative emotions, such as depression, indignation, and frustration, which may subsequently engender high psychological stress. This may force the poor to work harder to improve their family conditions as much as possible to narrow the wealth gap. However, owing to the lack of wealth among the poor, it is difficult to accumulate wealth through capital, and most of them can only increase their household income through labor. Some individuals may also overwork to narrow the wealth gap with other households. Thus, household wealth may reduce individuals’ labor supply.

A reduction in labor supply can increase an individuals’ mental health. Compared to individuals with relatively high wealth levels, those who lack accumulated wealth usually need to spend more energy on unpaid household labor and repetitive labor with pay, which may have a negative impact on their health [32,33]. Meanwhile, when individual labor hours are reduced, individuals tend to gain more leisure time. Such residents can spend their time engaging in some of their desired activities without the constraints of work. For example, individuals can spend their free time on sports, lunch breaks, coffee, socializing, and traveling. Positive social interactions (e.g., feeling protected and cared for) can make individuals feel happy and relaxed. Activities such as vacations and lunch breaks provide residents with good rest opportunities, thereby further making individuals feel good, giving them positive emotions, and reducing their stress levels [34]. In summary, the decrease in labor time and the increase in leisure time may both have a significant positive effect on residents’ mental health. Therefore, this study proposes the following hypothesis:

**Hypothesis** **3.** *Household wealth can improve individuals’ mental health by reducing individuals’ labor supply.*

## 3. Materials and Methods

### 3.1. Data

The data were mainly obtained from the China Family Panel Studies (CFPS) [35] undertaken by the China Social Science Survey Center (CSSSC) at Peking University. The baseline survey was officially launched by the CSSSC of Peking University in 2010, and data from the CFPS are now publicly available for 2010, 2012, 2014, 2016, 2018, and 2020 (given that the 2020 CFPS does not release household-related data, this study does not use the 2020 CFPS data). The CFPS is a nationally representative social-tracking survey. The sample covers most provinces in China. In terms of sampling content, the CFPS has conducted a lifelong follow-up of the individual samples and their households. The questionnaire not only asks individuals about their health status, marital status, gender, household registration, education level, and physical status, but also provides detailed household-level information, including the households’ financial assets, non-financial assets, liabilities, size, and business status. The effective sample was 72,196 after deleting observations with missing information.

### 3.2. Definition of Variables

Dependent variable. In this study, the mental health of individuals was measured using the Center for Epidemiologic Studies Depression (CES-D) scale in the CFPS data. The CES-D scale, developed by Radloff [36], is one of the most widely used instruments to measure mental health, and it is extensively applied in large surveys, such as the National Health and Nutrition Examination Survey and Health and Retirement Study. At present, many scholars have measured the mental health of individuals based on the CES-D scale [7,37,38,39,40]. The 2010 data published by the CFPS contain only six questions on the CES-D scale. To comprehensively appraise the respondents’ mental health, the 2012 data published by the CFPS included a 20-question CES-D scale. However, the feedback from the questionnaire interviewers indicated that the questions were not well accepted by respondents because of the multiplicity of the questions that needed to be answered. Therefore, as in 2010, the 2014 CFPS asked respondents only six relevant questions. To balance respondent acceptance and accuracy, the 2016 and 2018 CFPS used a streamlined eight-question CES-D scale to measure individuals’ mental health. Given that there are only six-question CES-D scales in 2010 and 2014 instead of the complete eight-question CES-D scales, such as in the years 2012, 2016, and 2018, this study only used data from the latter years when examining the effect of household wealth on residents’ mental health. Comparing the former years with the latter periods, the use of the eight-question CES-D scale provides a more comprehensive measure of an individuals’ mental health. The eight-question CES-D scale measures the mental health of individuals by selecting eight questions about mental health. The questionnaire of the CFPS asked eight questions about the frequency of the following feelings or behaviors over the past week: (1) I feel depressed; (2) I feel like I struggle to do anything; (3) I don’t sleep well; (4) I feel happy; (5) I feel lonely; (6) I live a happy life; (7) I feel sad and upset; and (8) I feel like I can’t get on with my life. Respondents could answer: a. hardly ever (less than one day); b. some of the time (1–2 days); c. often (3–4 days); d. most of the time (5–7 days). For questions (1), (2), (3), (5), (7), and (8), the a, b, c, and d responses were assigned the values 4, 3, 2, and 1, respectively. The a, b, c, and d responses to questions (4) and (6) were assigned the values 1, 2, 3, and 4, respectively. The above-stated transformation ensures that the higher the respondents’ score, the better the individuals’ mental health. Referring to Zhang et al. [7], the individual mental health status information was obtained by summing each sub-variable, with higher scores indicating better individuals’ mental health (factor analysis and principal component analysis were used to measure the mental health scores of individuals as robustness checks).

Key independent variable. Household wealth was the key explanatory variable. Referring to previous studies, this study used household net worth to measure the level of household wealth [41,42]. The CFPS questionnaire asked individual the information about the value of their property, household cash deposits, and household financial assets, such as stocks, funds, bonds, gold, financial derivatives, money owed by relatives and friends, and other wealth-related question. Moreover, the CFPS questionnaire contains information on household property liabilities, loans, and other borrowing-related information. The net wealth of the household was obtained by adding the household financial and non-financial assets and then subtracting the liabilities. It is worth noting that this study did not include household appliances, automobiles, or wealth, which are difficult to quantify numerically. Given that inflation may affect the estimation results, the consumer price index was used to deflate household wealth in the subsequent analysis, and the baseline year for the consumer price was 2012.

Control variables. As omitted variables can lead to endogeneity problems, control variables should be included when estimating the effects of household wealth on residents’ mental health. We adopted some control variables at the individual, household, and provincial levels. Specifically, this study controlled for individual characteristic variables such as gender (given that gender does not change over time, it was included in the individual fixed effect. Hence, we did not report the coefficient of gender in the following analysis), age, education level, marital status, household registration, work status, and smoking status. For household characteristic variables, this study controlled for household size. Regarding economic characteristics at the provincial level, this study controlled for the level of economic development, and we used the province-level per capital GDP as a proxy variable for the economic development level (given that the CFPS does not offer the exact information regarding which county the family lives in, this study only matched the province-level economic development level with the information of individuals). Considering that digital finance, as an important financial infrastructure, may promote the development of digital healthcare, reduce the cost of medical care for residents, and affect individuals’ health in many ways, we further controlled for the development status of digital inclusive finance. Referring to Guo et al. [43], this study used the Digital Inclusive Finance Index published by Peking University to measure the development status of digital inclusive finance. Given that the large values of the economic development level and digital inclusive finance, this study performed a logarithmic treatment of the economic development level and digital inclusive finance.

Table 1 reports the results of the descriptive statistical analysis of the explanatory variables (individual mental health and household wealth), as well as the control variables. The results of the descriptive statistical analysis in Table 1 show that the mean psychological health score is 26.993. The mean value of household net wealth is 3.902 (390,200 yuan). The mean value of household registration is 0.470, indicating that approximately 47% of the residents are urban, with equal percentages of urban and rural residents. The mean value of work status is 0.699, which indicates that approximately 70% of the individuals have their own jobs. The mean value of educational attainment is 7.045, indicating that the overall education level of our residents is not high.

### 3.3. Econometric Model

In this study, a two-way fixed effects model was used to examine the effects of household wealth on individuals’ mental health. The specific model setting is presented as follows:

(1) $$y_{it}=\alpha +\lambda wealth_{it}+\beta X+u_{i}+\gamma_{t}+\varepsilon_{it}$$

In the model, $y_{it}\;$represents the dependent variable mental health; α indicates the constant term;$\;wealth_{it}\;$implies the core explanatory variable, household net wealth; λ refers to the estimated coefficient of the effect of household wealth on mental health; and X is the control variable of individuals’ age, marital status, education level, household registration, work status, whether or not the individual smokes, household size, level of economic development, and digital financial inclusion development. β represents the vector of the estimated coefficients of the control variables, $u_{i}\;$ is the error terms that does not vary over time, $\gamma_{t}\;$ implies time effect, and $\varepsilon_{it}\;$ represents the random error terms that vary both over time and among individuals.

## 4. Results

### 4.1. Baseline Regression Results

Table 2 presents the results of the impact of household wealth on individuals’ mental health and their quadratic effects. Columns (1)–(3) report the effects of household wealth on individuals’ mental health. The estimation results in Column (1) indicate that the estimated coefficients of the effect of household wealth on residents’ mental health are positively significant without controlling for the covariate. Column (2) shows that, after accounting for the covariate, the estimated coefficient of household wealth remains positive and significant, which indicates that household wealth can have a significant positive effect on residents’ mental health. These results support Hypothesis 1. The estimated coefficient of squared household wealth in Column (3) is −0.001, which is negatively significant at the 1% significance level, indicating that the effect of household wealth on residents’ mental health is nonlinear but inverted U-shaped. The estimated results of the control variables in Column (2) show that the estimated coefficients of the effects of family size, marital status, job ownership, and regional economic development level on residents’ mental health are positively significant. The effects of urban household registration and smoking on residents’ mental health are negatively significant.

### 4.2. Considering Endogeneity

Endogeneity may exist when estimating the impact of household wealth on residents’ mental health. Although household wealth may affect individuals’ mental health through different channels, individuals’ mental health also affects household income and expenditure, which consequently affect household wealth, thereby creating a reciprocal causality problem. In addition, the problem of omitted variables caused by individuals’ overestimation or underestimation of their health levels may make this study biased in estimating the effect of household wealth on residents’ mental health. The Durbin–Wu–Hausman (DWH) test, which is also applicable in the presence of heteroskedasticity, is used to test the endogeneity of household wealth. The *p*-value of the DWH test is less than 0.001, indicating that household wealth is endogenous. To alleviate the endogeneity problem in estimating the causal relationship between household wealth and residents’ health, this study used two-stage least squares (2SLS) to estimate the effects of household wealth on residents’ mental health.

To use 2SLS for causal inference, it is necessary to determine the instrumental variables for household wealth. The instrumental variables need to ensure a strong correlation with household wealth, that is, there is no weak instrumental variable problem. In addition, the instrumental variables must satisfy exogeneity. To select instrumental variables, this study refers to Bucher-Koenen and Lusardi [44], who selected the mean wealth level of households other than one’s own household within the same village/household as the instrumental variable of household wealth. Individual household wealth may be influenced by the wealth of other households in the same village/household, but the mean value of the wealth level of other households in the same village/household is difficult to control for a particular individual or household. Hence, the mean value of the wealth level of other households in the same village/household is more exogenous than that of a particular household. In summary, it is reasonable for the selection of instrumental variables.

To test whether there is a weak instrumental variable problem, the Wald test with a nominal significance level of 5% was conducted. The “minimum characteristic statistic” of the Wald test is 43,276.6, which is much larger than the critical value of 8.96, indicating that there is no weak instrumental variable problem. Meanwhile, the F-value of the first stage of the 2SLS is 1819.36, which is much greater than the critical value of 10. The estimation results in Column (1) of Table 3 indicate that the coefficient of the first-stage instrumental variable is significantly positive, which indicates that the instrumental variable is not weak.

Table 3 reports the estimated results of the impact of household wealth on residents’ mental health, as obtained from 2SLS estimation. The results show that the estimated coefficient of the effect of household wealth on residents’ mental health is 0.0121 after accounting for endogeneity, which is positively significant. This indicates that household wealth has a significant positive impact on residents’ mental health even after considering endogeneity. This study used the results in Column (2) of Table 3, which considers endogeneity, as the baseline regression results and further examines the robustness, heterogeneity, and mechanism of the impact of household wealth on residents’ mental health.

### 4.3. Robustness Tests

To test the robustness of the effect of household wealth on residents’ mental health, corresponding robustness tests were conducted.

First, we control for province–time joint fixed effects. The foregoing context controls for time effects when estimating the impact of household wealth on residents’ mental health. Considering that some factors that change over time in some provinces may also have an impact on the mental health of the population, this study further controlled for province–time joint fixed effects. The results reported in Column (1) of Table 4 indicate that the estimated results of the effect of household wealth on residents’ mental health remain positively significant, thereby further implying a significant positive effect of household wealth on residents’ mental health.

Second, we used different methods to measure mental health. The foregoing context of individuals’ mental health status was evaluated by directly summing the individual scores of each sub-index of mental health status. To mitigate the measurement errors associated with the direct summation of the subscales, factor analysis with maximum likelihood estimation and principal component analysis were used to measure the mental health scores of individuals. Before factor analysis and principal component analysis, the Kaiser–Meyer–Olkin (KMO) test was performed in this study, and the value of the KMO was greater than 0.8 for both the factor analysis and principal component analysis. The results of Bartlett’s spherical test rejected the hypothesis of no correlation between variables at the 1% significance level, and Cronbach’s alpha was greater than 0.7. These results indicate that factor analysis and principal component analysis are appropriate for measuring individuals’ mental health. Principal component and factor analysis can be used to obtain the mental health score of residents. The higher the index value, the better the residents’ mental health. Columns (2) and (3) of Table 4 present the estimated results. The estimated coefficients of the impact of household wealth on individuals’ mental health are 0.0024 and 0.0025 in Columns (2) and (3), respectively, which are positively significant. Those results ensure the robustness of the impact of household wealth on residents’ mental health.

### 4.4. Heterogeneity Analysis

Individuals with different educational backgrounds show significant differences in work, household social status, and social security. The economic development and cultural customs between the western, central, and eastern regions have significant differences, and the impact of household wealth on the populations’ mental health at different education levels and in different regions may be nonhomogeneous. Hence, we investigated the effects of household wealth on individuals’ mental health with different education levels and regions.

#### 4.4.1. Heterogeneity in Education Level

In this study, the sample was divided into low- and high-education groups (if the education level of an individual is greater than the mean value of the overall population’s education level, the individual is considered to have a higher education level; otherwise, the individual is considered to be in the low education level group). Furthermore, the effects of household wealth on the mental health of residents with different education levels were examined separately using the 2SLS method. The results are presented in Table 5. The estimated coefficients of the effect of household wealth on the mental health of the low-education group and highly educated residents are positively significant, indicating that household wealth has a significant positive effect on the mental health of low-educated individuals as well as highly educated residents. We used the bootstrap sampling (1000 times) to compare the size of the coefficient of household wealth in different group, and the *p*-value obtained by bootstrap sampling is less than 0.01, which indicate that the coefficient of household wealth in the low-educated group and highly educated group has significant difference, and the former is greater than the latter. By comparing the estimated coefficients of the two groups, it is evident that the coefficient of household wealth on the mental health of individuals with low levels of education are greater than that of individuals with higher levels of education.

#### 4.4.2. Geographical Heterogeneity

The sample was divided into two subsamples, western and east–central, and the effects of household wealth on the mental health of residents in different regions were examined separately. As shown by the estimated coefficients of household wealth in Table 6, the estimated coefficients of the effects of household wealth on residents’ mental health in the western region are positive and significant, indicating that household wealth has a significant positive effect on the mental health of residents in the western region. In addition, the estimated coefficients of household wealth on the mental health of residents in the eastern and central regions are positive and significant, and the *p*-value obtained by bootstrap sampling is less than 0.01, indicating that the coefficient of household wealth in the western region group is greater than the central and eastern group. In summary, it can be established that the impact of household wealth on the mental health of residents in the western region is greater than that in the central and eastern regions.

### 4.5. Mechanism Analysis

#### 4.5.1. The Mediating Effect of Health Insurance

To test Hypothesis 2, this study employed a mediation model to investigate the mediating effect of the purchase of residential health insurance. The CFPS question related to the purchase of residential health insurance is: “Which health insurance do you have?”, and respondents can answer “Don’t know “, “Free medical care”, “Urban workers’ medical insurance”, “Urban resident medical insurance”, “Supplementary medical insurance”, “New rural cooperative medical care”, or “None of the above”. The sample with the answer “Don’t know” was deleted from this study. In addition, if an individual answered “None of the above”, the resident was considered to have no health insurance, and the health insurance variable was assigned a value of 0, and 1 otherwise. Table 7 reports the results of the mediation analysis (given that Table 2 and Table 3 present the impact of household wealth on individuals’ mental health, we do not report the results of household wealth on individual’s mental health in Table 7 and Table 8). The results in Column (1) of Table 7 indicate that the estimated coefficient of household wealth is 0.0019, which is positively significant. This indicates that the higher the household wealth, the more likely individuals are to purchase health insurance. The results in Column (2) suggest that the coefficient of health insurance is positive and significant, indicating that health insurance has a positive effect on a population’s mental health. These results support Hypothesis 2.

#### 4.5.2. The Mediating Effect of Labor Supply

To test Hypothesis 3, this study examined the mediating effect of residents’ labor supply using a mediation model. The CFPS provides data on individuals’ working hours per week, and we ook the individuals’ working hours per week as the proxy variable of an individual’s labor supply. Accordingly, the larger the value, the more supply hours the individual labor (the question on individual work hours in CFPS is: “In the past 12 months, how many hours per week did you generally work at your job?”. Where the hours worked do not include lunch breaks, but include overtime hours). However, given that the 2012 CFPS does not contain data related to individual weekly working hours, the 2014 CFPS does not contain data related to the eight-item CES-D scale. Therefore, this study examined the impact of household wealth on residents’ labor supply based on the 2016–2018 CFPS. Given that the men in China retire at the age of 60 and women retire at 55, if we start the first grade at the age of 6, we can graduate from college at 22. Residents may not participate in the labor market when they retire and while they are in college. Therefore, to ensure the robustness of the mechanism analysis, this study divided the sample into 2 subsamples of females aged from 22 to 56 and males aged from 22 to 61 to investigate the effects of household wealth on individuals’ labor supply by using a mediation model. The results are presented in Table 8. 

The estimated coefficient of household wealth in Column (1) is −0.1407, which is negatively significant at the 1% significance level. This indicates that household wealth has a significant negative impact on individuals’ labor supply, and the coefficient of work hours in Column (2) is negatively significant, indicating that work hours have a negative impact on individuals’ mental health. These results support Hypothesis 3. Meanwhile, the results in Columns (3)–(6) indicate that the mediating effects of labor supply on mental health for both women and men are significant. These findings suggest that household wealth can improve mental health by reducing individuals’ labor supply.

### 4.6. Further Analysis

The current impact of household wealth on residents’ mental health was examined above, and this study further explored the medium- and long-term impacts of household wealth on residents’ mental health. We also controlled for changes in household wealth, as they may impact mental health. As the CFPS is researched every two years, to examine the medium- and long-term effects of household wealth on residents’ health, this study matched the mental health of residents in 2018 with some control variables in 2012. We examined the effect of the household wealth in 2012 on the residents’ mental health status in 2018, that is, the effects of household wealth on the residents’ mental health status six years later. The difference between household wealth in 2018 and 2012 reflects the six-year change at the household level. The higher the value, the more household wealth increased over the six years. Similarly, this study also examined the effect of household wealth and changes in household wealth on residents’ mental health after two and four years. 

The estimated results of the medium- and long-term effects of household wealth on residents’ health in Table 9 show that the estimated coefficient of household wealth in Column (1) is 0.0491, which is positively significant at the 1% significance level, indicating that household wealth has a significant positive effect on residents’ mental health. In addition, the estimated coefficients of the effects of household wealth on individuals’ mental health in Columns (2) and (3) of Table 9 are all positively significant. In summary, it can be established that household wealth has not only a positive short-term impact on residents’ mental health but also a significant positive impact on residents’ mental health in the medium and long terms. The estimated coefficient of the change in household wealth in Columns (1) to (3) is positively significant at the 1% level, indicating that an increase in household wealth has a positive impact on individuals’ mental health.

## 5. Discussion

Several studies have examined the effects of household wealth on individuals’ mental health, and most of them have concluded that household wealth has a significant positive effect on mental health [9,38,45,46]. Mclnemey et al. [38] indicate that sudden wealth losses have a significant negative impact on individuals’ mental health, but they do not investigate the mechanism of the effects of wealth losses on mental health. Yilmazer et al. [9] report that stress and negative changes in health-related behaviors, such as expenditure on health care, are the mechanisms that link wealth and individuals’ mental health. Galama and Van Kippersluis [47] indicate that wealth can affect an individuals’ health through its effect on healthy consumption. In addition, previous studies have revealed that labor supply and leisure choices are important factors affecting mental health [45]. However, some studies have also suggested that wealth cannot affect residents’ health [48,49]. For example, Östling et al. [48] used survey data collected by Statistics Sweden to investigate the impact of unearned wealth from lotteries on individuals’ health and found that there were no statistically significant associations between unearned wealth and individuals’ mental health. Lindqvist et al. [49] find that the impact of a positive wealth shock on Swedish residents’ mental health is not significant. One possible explanation is that in Western European countries, such as Sweden and Germany, residents have adequate social security, and an unexpected increase in wealth hardly affects individuals’ mental health. Therefore, no consistent conclusions have been reached regarding the effects of wealth on residents’ mental health.

Some studies have assessed the impact of wealth on health using cross-sectional data [46,50,51]. For example, Park et al. [50] examined the effects of household wealth on physical health but did not investigate the effect of wealth on mental health, and the study was conducted with an older population. Kumar et al. [51] found that household wealth had a positive impact on individual mental health. Ettman et al. [46] examined the impact of household wealth on individuals’ mental health and found a significant positive relationship between household wealth and mental health. However, it is difficult to consider the heterogeneity of individuals because cross-sectional data can only roughly test whether individuals with higher levels of wealth have better health. Individual heterogeneity, such as personality, IQ, or genetic differences, may confound the effects of wealth on health. In other words, what is obtained from the cross-sectional data may only reflect the correlation between wealth and health, and it is difficult to identify the causal relationship between household wealth and mental health. Therefore, unlike the abovementioned studies, this study used the 2012–2018 CFPS panel data to identify the causal relationship between household wealth and individuals’ mental health and to examine the mechanism of the effect of household wealth on mental health. The results of this study are more accurate compared with the cross-sectional studies.

Notably, several studies have examined the impact of household wealth on health based on panel data [18,52,53,54]. For example, Jou et al. [54] examined the impact of housing wealth on individuals’ mental health based on the panel study of income dynamics data and found that property wealth has a significant positive impact on individuals’ self-rated mental health. In contrast, this study did not focus on property wealth but examined the effects of household net wealth on individuals’ mental health. Additionally, this study examined both the linear and nonlinear effects of wealth on individuals’ psychological health. Additionally, some studies have examined the impact of household wealth on Chinese residents’ health. For example, Xu and Xie [53] examined the impact of household wealth on residents’ physical health using CFPS panel data for 2010 and 2012. However, the study did not examine the effects of household wealth on residents’ mental health, neither did it consider the endogeneity between household wealth and individuals’ health nor the mechanisms by which household wealth affected residents’ health. Unlike Xu and Xie [53], this study examined the effects of household wealth on individuals’ mental health rather than the impact of wealth on physical health. In addition, this study considered endogeneity and examines the mechanism of the effects of household wealth on individuals’ mental health.

Other studies have also examined the causal relationship between wealth shocks and population health based on “quasi-natural experiments” in which lottery winnings, stock market booms, financial crises, and estate taxes may exogenously shock household wealth [15,40,55,56,57,58,59]. Most studies show that positive wealth shocks have a significant positive impact on individuals’ health. Unlike the abovementioned studies, this study examined the impact of cumulative household wealth on individuals’ mental health. In terms of research content, most previous studies have only examined the linear effects of wealth on health [46,54], and merely a few studies have examined the nonlinear effects of wealth on health. Given that the impact of wealth on residents’ health may be nonlinear, this study examined the linear and nonlinear impact of household wealth on residents’ mental health.

The results of this study suggest that household wealth significantly improves individuals’ mental health. This finding is consistent with those reported by Kumar et al. [51], Ettman et al. [46], and Jou et al. [54]. However, unlike these studies, our study examined the effects of household wealth on individuals’ mental health not only in the short term but also in the medium and long terms. We found that household wealth has positive medium- and long-term impacts on individuals’ mental health and that changes in wealth have a significant impact on mental health. In addition, this study found that the effects of household wealth on psychological health is not linear but has an inverted U-shaped relationship, which is in line with the results of Hurd and Kapteyn [59]. The possible explanation for the inverted U-shaped relationship between household wealth and individuals’ mental health is that, to accumulate more wealth, some populations might overwork or invest in high-risk and high-return financial products. This causes individuals to lack rest or leisure and increases their pressure, which affects their mental health [60]. The results of the mechanism analysis suggest that household wealth encourages individuals or families to invest in health insurance, which agrees with the findings of Bernard et al. [61]. Furthermore, the effect of household wealth on labor supply is negatively significant for both females and males, which is consistent with the results of previous studies [62,63].

In addition, this study established that the effects of household wealth on the mental health of individuals with different levels of education across regions is nonhomogeneous. The results of the heterogeneity analysis indicate that the effects of household wealth on the mental health of individuals with lower levels of education is greater. This result is consistent with the findings of Raschke [64]. This may be attributed to the fact that residents with low levels of education have much lower incomes and wealth accumulation than those with higher levels of education. An increase in the same level of wealth may result in greater utility gains for less educated individuals than for more educated individuals. Therefore, the effects of household wealth on the mental health of less educated individuals are greater. In addition, the impact of household wealth on the psychological health of individuals in Western China is greater. This is because the western region is lower than the eastern and central regions, both in terms of infrastructure construction and level of economic development. Meanwhile, housing prices in the eastern region are, on average, higher than those in the western region. Consequently, residents in the eastern and central regions are more affluent overall than those in the western region. According to the law of diminishing marginal utility, as the level of wealth increases, the utility of each unit of wealth increase is gradually reduced, and the same unit of wealth increase brings more satisfaction to relatively poor residents in the western region than to those in the eastern and central regions.

The limitations of this study are mainly reflected in the following aspects. First, we tried to control for some control variables that may affect both household wealth and individuals’ mental health, but we could not control for some control variables that are difficult to measure, that is, there are missing variables. Second, owing to space and data constraints, we did not investigate the mechanism of the inverted U-shaped relationship between household wealth and psychological health through empirical analysis. Third, although we attempted to select instrumental variables for household wealth and estimate the causal relationship between household wealth and individuals’ mental health using 2SLS, the instrumental variables selected in this study may not be completely exogenous. Future research could try to find more exogenous instrumental variables as instrumental variables of household wealth to better identify the causal relationship between household wealth and individuals’ mental health. In addition, future research could examine the effects of exogenous wealth shocks on individuals’ mental health. Finally, in addition to absolute wealth, the impact of relative household wealth (household wealth gap) on individuals’ mental health is an important and interesting issue that can be addressed in the future.

This study has the following policy implications. In the context of the COVID-19 pandemic, individuals face enormous challenges in terms of their physical and mental health status. Wealth can provide individuals with a sense of security as well as basic living security and medical insurance. Improving household wealth can also enhance individuals’ mental health. Given that income is one of the main sources of household wealth accumulation, the government should increase the income of residents and broaden their sources of income. In addition, the mechanism analysis in this study reveals that household wealth can influence individuals’ mental health by increasing household insurance investment and reducing individuals’ labor supply. Therefore, the government should encourage residents to invest in health insurance and provide a certain amount of cash subsidy to families who cannot pay premiums due to a lack of funds, thus increasing their willingness to pay for health insurance. Finally, enterprises should create a good working atmosphere, provide workers with sufficient rest time, and avoid ineffective overtime work.

## 6. Conclusions

Based on nationwide representative survey data, this study examined the causal relationship between household wealth and individuals’ mental health. The results reveal that household wealth significantly improves individuals’ mental health. Another important finding of this study is the inverted U-shaped relationship between household wealth and individuals’ psychological health. This study also examined the heterogeneity of the effects of household wealth on the mental health of individuals with different levels of education and in different regions. The heterogeneity analysis results indicate that household wealth has a greater impact on the mental health of low-educated groups and individuals in the western region. To further clarify the mechanism of the effect of household wealth on individuals’ mental health, this study also conducted a mechanism of action test. The results show that household wealth can influence individuals’ mental health by encouraging individuals or families to purchase health insurance and reducing individuals’ labor supply. Finally, this study further investigated the medium- and long-term effects of household wealth on individuals’ mental health and established that household wealth affects not only individuals’ mental health in the present but also their mental health in the medium and long terms.

## Figures and Tables

**Table 1:** Definition of variables.

Variable	Definition	Observation	Mean	SD
Mental health	A comprehensive index aggregated by 8-question CES-D scale indexes, with higher scores indicating greater mental health.	72,196	26.993	3.902
Wealth	Net wealth of the family	72,196	3.902	6.599
Age	Age of the respondent	72,196	55.528	19.566
Size	Number of family members	72,196	4.285	1.997
Marry	1 for married, 0 otherwise	72,196	0.840	0.366
Urban	1 for urban *hukou*, 0 otherwise	72,196	0.470	0.499
Work	1 for worker (whether self-employed or employed), 0 otherwise	72,196	0.699	0.459
Smoke	1 for smoker, 0 otherwise	72,196	0.301	0.459
Education	Years of education	72,196	7.045	5.005
GDP	Per capital GDP on the provincial level	72,196	10.755	0.426
F_index	The index of digital inclusive finance	72,196	5.254	0.481

**Table 2:** Baseline estimation results.

Variable	(1)	(2)	(3)
	Mental Health	Mental Health	Mental Health
Wealth	0.012 ***	0.009 **	0.035 ***
	(0.004)	(0.004)	(0.010)
Wealth × Wealth			−0.001 ***
			(0.000)
Age		−0.003	−0.003
		(0.004)	(0.004)
Size		0.052 ***	0.051 ***
		(0.016)	(0.016)
Marry		0.979 ***	0.978 ***
		(0.105)	(0.105)
Urban		−0.241 ***	−0.252 ***
		(0.088)	(0.088)
Work		0.198 ***	0.199 ***
		(0.050)	(0.050)
Smoke		−0.175 **	−0.175 **
		(0.081)	(0.081)
Education		−0.006	−0.007
		(0.021)	(0.021)
GDP		0.779 ***	0.774 ***
		(0.161)	(0.160)
F_index		−0.039	−0.097
		(0.248)	(0.250)
Individual fixed effect	YES	YES	YES
Year fixed effect	YES	YES	YES
Constant	27.293 ***	18.531 ***	18.819 ***
	(0.023)	(1.846)	(1.850)
Observation	72196	72196	72196
R2	0.034	0.040	0.040

Note: ** *p* < 0.05, *** *p* < 0.01. Standard errors are reported in parentheses.

**Table 3:** 2SLS estimation results.

Variable	(1)	(2)
	First-Stage Results	Second-Stage Results
Wealth		0.0121 ***
		(0.0043)
IV	0.7063 ***	
	(0.0081)	
Control variable	YES	YES
Year fixed effect	YES	YES
Constant	−20.0875 ***	15.9221 ***
	(0.9858)	(0.6349)
Observation	72196	72196
R2	0.5494	0.0725

Note: *** *p* < 0.01. Standard errors are reported in parentheses.

**Table 4:** Results of the robustness checks.

Variable	(1)	(2)	(3)
	Mental Health	Mental Health	Mental Health
Wealth	0.0483 ***	0.0024 ***	0.0025 ***
	(0.0061)	(0.0007)	(0.0009)
Control variable	YES	YES	YES
Year fix effect	YES	YES	YES
Province × Year fixed effect	YES	NO	NO
Constant	−7.0976 **	−1.6901 ***	−2.0839 ***
	(3.0407)	(0.1143)	(0.1324)
Observation	72196	72196	72196
R2	0.089	0.059	0.063

Note: ** *p* < 0.05, *** *p* < 0.01. The parentheses reported the standard errors.

**Table 5:** Heterogenous effect with respect to the different education levels.

Variable	(1)	(2)
	Low-Educated	High-Educated
Wealth	0.046 ***	0.014 ***
	(0.010)	(0.005)
Control variable	YES	YES
Year fixed effect	YES	YES
Constant	13.011 ***	20.164 ***
	(1.035)	(0.797)
Observation	34492	37704
R2	0.067	0.041

Note: *** *p* < 0.01. The parentheses reported the standard errors.

**Table 6:** Heterogenous effect with respect to regions.

Variable	(1)	(2)
	Western Region	East and Central Region
Wealth	0.1042 ***	0.0293 ***
	(0.0191)	(0.0048)
Control variable	YES	YES
Year fixed effect	YES	YES
Constant	−1.1075	23.5909 ***
	(1.7786)	(0.8358)
Observation	24248	47948
R2	0.076	0.058

Note: *** *p* < 0.01. The parentheses reported the standard errors.

**Table 7:** Mediating effect of health insurance.

Variable	(1)	(2)
	Insurance	Mental Health
Wealth	0.0019 ***	0.0231 ***
	(0.0002)	(0.0025)
Insurance		0.3529 ***
		(0.0496)
Control variables	YES	YES
Year fixed effect	YES	YES
Constant	YES	YES
Observation	71976	71976

Note: *** *p* < 0.01. The parentheses reported the standard errors.

**Table 8:** Mediating effect of resident’s labor supply.

Variable	(1)	(2)	(3)	(4)	(5)	(6)
	Full Sample		Female (22 < Age < 56)		Male (22 < Age < 61)	
	Work Hour	Mental Health	Work Hour	Mental Health	Work Hour	Mental Health
Wealth	−0.1407 ***	0.0220 ***	−0.1196 ***	0.0136 **	−0.1326 ***	0.0190 ***
	(0.0236)	(0.0037)	(0.0433)	(0.0065)	(0.0402)	(0.0059)
Work hour		−0.0045 ***		−0.0042 ***		−0.0051 ***
		(0.0009)		(0.0015)		(0.0014)
Control variables	YES	YES	YES	YES	YES	YES
Year fixed effect	YES	YES	YES	YES	YES	YES
Constant	YES	YES	YES	YES	YES	YES
Observation	30702	30702	9945	9945	11274	11274

Note: ** *p* < 0.05, *** *p* < 0.01. The parentheses reported the standard errors.

**Table 9:** The further analysis results.

Variable	(1)	(2)	(3)
	Mental Health	Mental Health	Mental Health
Lagged 6 years’ wealth	0.0491 ***		
	(0.0084)		
Lagged 4 years’ wealth		0.0369 ***	
		(0.0051)	
Lagged 2 years’ wealth			0.0171 ***
			(0.0046)
Δwealth (6 year)	0.0234 ***		
	(0.0071)		
Δwealth (4 year)		0.0171 ***	
		(0.0046)	
Δwealth (2 year)			0.0102 **
			(0.0043)
Control variable	YES	YES	YES
Constant	YES	YES	YES
Observation	13049	29403	33918
R2	0.0656	0.0722	0.0712

Note: ** *p* < 0.05, *** *p* < 0.01. The parentheses reported the standard errors.

## Data Availability

The data used in this study can be found in the China Family Panel Survey, http://www.isss.pku.edu.cn/cfps/ (accessed on 28 July 2022).

## References

[1] Ojeda V.D., Frank R.G., McGuire T.G., Gilmer T.P. (2010). Mental illness, nativity, gender and labor supply. Health Econ..

[2] Evans S., Banerjee S., Leese M., Huxley P. (2007). The impact of mental illness on quality of life: A comparison of severe mental illness, common mental disorder and healthy population samples. Qual. Life Res..

[3] Suicide. https://www.who.int/news-room/fact-sheets/detail/suicide.

[4] Carleton T.A. (2017). Crop-damaging temperatures increase suicide rates in India. Proc. Natl. Acad. Sci. USA.

[5] Obradovich N., Migliorini R., Paulus M.P., Rahwan I. (2018). Empirical evidence of mental health risks posed by climate change. Proc. Natl. Acad. Sci. USA.

[6] Choi K.H., Bae S., Kim S., Kwon H.J. (2020). Indoor and outdoor PM2. 5 exposure, and anxiety among schoolchildren in Korea: A panel study. Environ. Sci. Pollut. Res..

[7] Zhang R., Zhang Y., Dai Z. (2022). Impact of natural disasters on mental health: A cross-sectional study based on the 2014 China Family Panel Survey. Int. J. Environ. Res. Public Health.

[8] Bartoll X., Palència L., Malmusi D., Suhrcke M., Borrell C. (2014). The evolution of mental health in Spain during the economic crisis. Eur. J. Public Health.

[9] Yilmazer T., Babiarz P., Liu F. (2015). The impact of diminished housing wealth on health in the United States: Evidence from the Great Recession. Soc. Sci. Med..

[10] Achim M.V., Văidean V.L., Borlea S.N. (2020). Corruption and health outcomes within an economic and cultural framework. Eur. J. Health Econ..

[11] Chirico F. (2017). Religious belief and mental health in lay and consecrated Italian teachers. J. Relig. Health.

[12] Dai X., Gu N. (2021). The impact of social capital on mental health: Evidence from the China Family Panel Survey. Int. J. Environ. Res. Public Health.

[13] Schaller J., Stevens A.H. (2015). Short-run effects of job loss on health conditions, health insurance, and health care utilization. J. Health Econ..

[14] Ettner S.L. (1996). New evidence on the relationship between income and health. J. Health Econ..

[15] Frijters P., Haisken-DeNew J.P., Shields M.A. (2005). The causal effect of income on health: Evidence from German reunification. J. Health Econ..

[16] Silbersdorff A., Lynch J., Klasen S., Kneib T. (2018). Reconsidering the income-health relationship using distributional regression. Health Econ..

[17] Kim S., Koh K. (2021). The effects of income on health: Evidence from lottery wins in Singapore. J. Health Econ..

[18] Hajat A., Kaufman J.S., Rose K.M., Siddiqi A., Thomas J.C. (2010). Do the wealthy have a health advantage? Cardiovascular disease risk factors and wealth. Soc. Sci. Med..

[19] Pollack C.E., Cubbin C., Sania A., Hayward M., Vallone D., Flaherty B., Braveman P.A. (2013). Do wealth disparities contribute to health disparities within racial/ethnic groups?. J. Epidemiol. Community Health.

[20] Braveman P.A., Cubbin C., Egerter S., Chideya S., Marchi K.S., Metzler M., Posner S. (2005). Socioeconomic status in health research: One size does not fit all. Jama.

[21] Pollack C.E., Chideya S., Cubbin C., Williams B., Dekker M., Braveman P. (2007). Should health studies measure wealth? A systematic review. Am. J. Prev. Med..

[22] Adler N.E., Rehkopf D.H. (2008). US disparities in health: Descriptions, causes, and mechanisms. Annu. Rev. Public Health.

[23] Schunck R. (2013). Within and between estimates in random-effects models: Advantages and drawbacks of correlated random effects and hybrid models. Stata J..

[24] McEwen B.S. (1998). Stress, adaptation, and disease: Allostasis and allostatic load. Ann. N. Y. Acad. Sci..

[25] Schneiderman N., Ironson G., Siegel S.D. (2005). Stress and health: Psychological, behavioral, and biological determinants. Annu. Rev. Clin. Psychol..

[26] Lynch J.W., Smith G.D., Kaplan G.A., House J.S. (2000). Income inequality and mortality: Importance to health of individual income, psychosocial environment, or material conditions. Bmj.

[27] Williams D.R., Collins C. (2016). Racial residential segregation: A fundamental cause of racial disparities in health. Public Health Rep..

[28] Subramanian S.V., Kawachi I. (2004). Income inequality and health: What have we learned so far?. Epidemiol. Rev..

[29] Eibner C., Evans W.N. (2005). Relative deprivation, poor health habits, and mortality. J. Hum. Resour..

[30] Finkelstein A., Taubman S., Wright B., Bernstein M., Gruber J., Newhouse J.P., Allen H., Baicker K., Oregon Health Study Group (2012). The Oregon health insurance experiment: Evidence from the first year. Q. J. Econ..

[31] Woolhandler S., Himmelstein D.U. (2017). The relationship of health insurance and mortality: Is lack of insurance deadly?. Ann. Intern. Med..

[32] Burgard S.A., Ailshire J.A., Kalousova L. (2013). The Great Recession and health: People, populations, and disparities. Ann. Am. Acad. Political Soc. Sci..

[33] Boen C., Keister L., Aronson B. (2020). Beyond net worth: Racial differences in wealth portfolios and black–white health inequality across the life course. J. Health Soc. Behav..

[34] Pressman S.D., Matthews K.A., Cohen S., Martire L.M., Scheier M., Baum A., Schulz R. (2009). Association of enjoyable leisure activities with psychological and physical well-being. Psychosom. Med..

[35] China Family Panel Studies. http://www.isss.pku.edu.cn/cfps/.

[36] Radloff L.S. (1991). The Use of the Center for Epidemiologic Studies Depression Scale in Adolescents and Young Adults. J. Youth Adolesc..

[37] Kim B., Ruhm C.J. (2012). Inheritances, health and death. Health Econ..

[38] McInerney M., Mellor J.M., Nicholas L.H. (2013). Recession depression: Mental health effects of the 2008 stock market crash. J. Health Econ..

[39] Marshall A., Jivraj S., Nazroo J., Tampubolon G., Vanhoutte B. (2014). Does the level of wealth inequality within an area influence the prevalence of depression amongst older people?. Health Place.

[40] Schwandt H. (2018). Wealth shocks and health outcomes: Evidence from stock market fluctuations. Am. Econ. J. Appl. Econ..

[41] Hurst E., Lusardi A. (2004). Liquidity constraints, household wealth, and entrepreneurship. J. Political Econ..

[42] Piketty T., Zucman G. (2014). Capital is back: Wealth-income ratios in rich countries 1700–2010. Q. J. Econ..

[43] Guo F., Wang J., Wang F., Kong T., Zhang X., Cheng Z. (2020). Measuring China’s Digital Financial Inclusion: Index Compilation and Spatial Characteristics. China Econ. Q..

[44] Bucher-Koenen T., Lusardi A. (2011). Financial literacy and retirement planning in Germany. J. Pension Econ. Financ..

[45] Fichera E., Gathergood J. (2016). Do wealth shocks affect health? New evidence from the housing boom. Health Econ..

[46] Ettman C.K., Cohen G.H., Galea S. (2020). Is wealth associated with depressive symptoms in the United States?. Ann. Epidemiol..

[47] Galama T.J., Van Kippersluis H. (2019). A theory of socio-economic disparities in health over the life cycle. Econ. J..

[48] Östling R., Cesarini D., Lindqvist E. (2020). Association between lottery prize size and self-reported health habits in Swedish lottery players. JAMA Netw. Open.

[49] Lindqvist E., Östling R., Cesarini D. (2020). Long-run effects of lottery wealth on psychological well-being. Rev. Econ. Stud..

[50] Park B.H., Jung M.S., Lee T.J. (2009). Associations of income and wealth with health status in the Korean elderly. J. Prev. Med. Public Health.

[51] Kumar K., Shukla A., Singh A., Ram F., Kowal P. (2016). Association between wealth and health among older adults in rural China and India. J. Econ. Ageing.

[52] Hajat A., Kaufman J.S., Rose K.M., Siddiqi A., Thomas J.C. (2011). Long-term effects of wealth on mortality and self-rated health status. Am. J. Epidemiol..

[53] Xu H., Xie Y. (2017). Socioeconomic inequalities in health in China: A reassessment with data from the 2010–2012 China family panel studies. Soc. Indic. Res..

[54] Jou A., Mas N., Vergara-Alert C. (2020). Housing Wealth, Health and Deaths of Despair. J. Real Estate Financ. Econ..

[55] Snyder S.E., Evans W.N. (2006). The effect of income on mortality: Evidence from the social security notch. Rev. Econ. Stat..

[56] Van Kippersluis H., Galama T.J. (2014). Wealth and health behavior: Testing the concept of a health cost. Eur. Econ. Rev..

[57] Apouey B., Clark A.E. (2015). Winning big but feeling no better? The effect of lottery prizes on physical and mental health. Health Econ..

[58] Erixson O. (2017). Health responses to a wealth shock: Evidence from a Swedish tax reform. J. Popul. Econ..

[59] Hurd M., Kapteyn A. (2003). Health, Wealth, and the Role of Institutions. J. Hum. Resour..

[60] Graham C., Zhou S., Zhang J. (2017). Happiness and health in China: The paradox of progress. World Dev..

[61] Bernard D.M., Banthin J.S., Encinosa W.E. (2009). Wealth, income, and the affordability of health insurance. Health Aff..

[62] Li F., Xiao J.J. (2020). Losing the future: Household wealth from urban housing demolition and children’s human capital in China. China Econ. Rev..

[63] Li H., Li J., Lu Y., Xie H. (2020). Housing wealth and labor supply: Evidence from a regression discontinuity design. J. Public Econ..

[64] Raschke C. (2019). Unexpected windfalls, education, and mental health: Evidence from lottery winners in Germany. Appl. Econ..

